# Diagnostic application of exome sequencing in Chinese children with suspected inherited kidney diseases

**DOI:** 10.3389/fgene.2022.933636

**Published:** 2023-01-06

**Authors:** Min Gao, Fengling Yu, Rui Dong, Kaihui Zhang, Yuqiang Lv, Jian Ma, Dong Wang, Hongxia Zhang, Zhongtao Gai, Yi Liu

**Affiliations:** ^1^ Pediatric Research Institute, Children’s Hospital Affiliated to Shandong University, Jinan, China; ^2^ Shandong Provincial Clinical Research Center for Children’s Health and Disease, Jinan, China; ^3^ Clinical Laboratory, Children’s Hospital Affiliated to Shandong University, Ji’nan, China; ^4^ Department of Nephrology, Children’s Hospital Affiliated to Shandong University, Jinan, China

**Keywords:** exome sequencing, inherited kidney diseases, clinical diagnosis, diagnostic utility, early diagnosis

## Abstract

**Background:** Inherited kidney diseases (IKDs) are a group of kidney diseases characterized by abnormal kidney structure or function caused by genetic factors, but they are not easily diagnosed in childhood due to either nonspecific symptoms and signs or clinically silent symptoms in the early stages until the progressive stages, even end-stages. Early diagnosis of IKDs is very urgent for timely treatment and improving outcomes of patients. So far, the etiological diagnosis has been accelerated with the advance of clinical genetic technology, particularly the advent of next-generation sequencing (NGS) that is not only a powerful tool for prompt and accurate diagnosis of IKDs but also gives therapy guidance to decrease the risk of unnecessary and harmful interventions.

**Methods:** The patients presenting with urinalysis abnormalities or structural abnormalities from 149 Chinese families were enrolled in this study. The clinical features of the patients were collected, and the potentially causative gene variants were detected using exome sequencing. The clinical diagnostic utility of the genetic testing was assessed after more detailed clinical data were analyzed.

**Result:** In total, 55 patients identified having causative variants by exome sequencing were genetically diagnosed, encompassing 16 (29.1%) autosomal dominant IKDs, 16 (29.1%) autosomal recessive IKDs, and 23 (41.8%) X-linked IKDs, with 25 unreported and 45 reported variants. The diagnostic yield was 36.9%. The utility of the exome sequencing was accessed, 12 patients (21.8%) were confirmed to have suspected IKDs, 26 patients (47.3%) discerned the specific sub-types of clinical category, and 17 patients (30.9%) with unknown etiology or lack of typical manifestations were reclassified.

**Conclusion:** Our study supported that genetic testing plays a crucial role in the early diagnosis for children with IKDs, which affected follow-up treatment and prognostic assessment in clinical practice. Moreover, the variant spectrum associated with IKDs was expanded.

## Introduction

Inherited kidney diseases (IKDs) are a group of kidney diseases characterized by abnormal kidney structure or function caused by genetic factors which present substantial clinical heterogeneity in clinical presentation, age of onset, severity, and progression of symptoms, accounting for at least 10%–15% of cases of kidney replacement therapy (Zhang et al., 2021; Torra et al., 2021). So far, there have been more than 150 known IKDs which can be divided into two categories of inherited renal structural abnormality and inherited renal dysfunction (Devuyst et al., 2014; Cunha et al., 2020).

IKDs are caused by different factors that can lead to permanent and irreversible deterioration of renal function, but they are not easily diagnosed in childhood due to either nonspecific symptoms and signs or clinically silent symptoms in the early stages until the progressive stages, even end-stages, when presenting characteristics of kidney impairment such as proteinuria and hematuria and extrarenal symptoms; thereby, early diagnosis of IKDs is very urgent for timely treatment and improving outcomes of patients. Renal biopsy has been the gold standard for diagnosis of kidney diseases since it was introduced in the 1950s by providing the injury pattern and prognosis information, while the limitation has become more and more obvious in the era of precision medicine as it is impossible to make an early, precise diagnosis of IKDs (Liapis and Gaut., 2013; Hogan et al., 2016; Murray et al., 2020). The etiological diagnosis has been accelerated with the advance of clinical genetic technology, particularly the advent of next-generation sequencing (NGS) that is not only a powerful tool for prompt and accurate diagnosis of IKDs but also gives therapy guidance to decrease the risk of unnecessary and harmful interventions (Groopman et al., 2019; Devarajan et al., 2022).

The genetic causes of more than 150 rare kidney diseases and variants in over 400 genes have been defined since the first case of a causal variant for a monogenic kidney disorder (Alport syndrome) was identified in 1990 (Barker et al., 1990; Knoers et al., 2022). NGS technology enabling the detection of all types of genetic variants and benefiting prompt and accurate diagnosis has been applied in detecting disease-causing genes of IKDs in children, but the data obtained from the clinical practice setting are rare, so recommendations have been made to use NGS techniques in the diagnosis of patients with kidney diseases (Groopman et al., 2019; Knoers et al., 2022).

Here, we present an investigation of causative gene variations in a well-characterized clinical cohort of 149 unrelated patients with urinalysis abnormalities or structural abnormalities from Shandong, a northern province in China, using exome sequencing. We identified 70 clinically diagnostic variants in 55 patients, and the diagnostic yield was 36.9%.

## Materials and methods

### Ethics statement

This work was approved by the Medical Ethics Committee of Children’s Hospital Affiliated to Shandong University. Informed consent was obtained from the patient’s parents before clinical and laboratory examinations were performed on the probands. The information of all the patients was anonymized prior to submission. All the procedures performed in the study were in accordance with the Declaration of Helsinki.

### Subjects

A total of 149 probands with clinical suspected IKDs or unexplained proteinuria or/and hematuria who were referred for NGS in our institute of Children’s Hospital Affiliated to Shandong University (CHASU) from September 2017 to December 2021 were enrolled in this study. Their parents, except four unavailable fathers, were also enrolled in the study. All probands from Shandong, a northern province in China, were clinically examined and diagnosed by experienced pediatric nephrologists at the Department of Nephrology in CHASU following the proposal that all patients had urinalysis abnormalities or structural abnormalities and excluded the following causes or conditions: trauma, infectious diseases, inflammatory processes, malignant tumors, metabolic disorders, bleeding and blood cell disorders, and vascular disorders.

### Exome sequencing

Genomic DNA was isolated from peripheral blood samples of the probands and their parents using the TIANamp Blood DNA Kit (TIANGEN, Beijing, China) following the manufacturer’s instructions and used for library preparation. The exon regions along with adjacent intron regions (±20 bp) of all genes contained in post-PCR libraries were then captured and enriched. Enriched sample libraries were quantified by Qubit dsDNA HS Assay (Thermal Fisher Scientific), and library size was checked by High Sensitivity D1000 Reagents (Agilent, United States). Final libraries were sequenced using the Human Exome Probes P039-Exome (MyGenostics, Beijing, China) on an Illumina NovaSeq 5000 sequencer (Illumina, United States).

### Variant analysis and validation

The sequencing data were compared to the UCSC hg19 human reference genome sequence using NextGene V2.3.4 software, and the genetic variation was identified. The quality parameters such as coverage of the target area and average sequencing depth were collected. Annotations were made using NextGene V2.3.4 software. Potential effects of the variants on function were assessed using the following databases: UCSC Genome Bioinformatics (http://genome.ucsc.edu/) (comparing with the reference sequence of SMARCA2 gene); biological function prediction (Sorting Intolerant From Tolerant (http://sift.jcvi.org), PolyPhen-2 (http://genetics.bwh.harvard.edu/pph2), Mutation Taster (http://www.mutationtaster.org/)), frequency of normal population (1000 Genomes (http://browser.1000genomes.org), Exome Aggregation Consortium (ExAC, http://exac.broadinstitute.org), dbSNP database, Human Gene Mutation Database (http://www.hgmd.cf.ac.uk), ClinVar (http://www.ncbi.nlm.nih.gov/clinvar), and OMIM databases. Genetic variation was further screened using the following steps: 1) prioritize public attention genetic variants that may be associated with disease in the database, small insertions/deletions (INDEL), typical splice site changes, and missense variations; 2) in the normal population, the secondary allele frequency is <5% (except for the known pathogenicity of MAF ≥ 5%); and 3) the genetic variation included in the HGMD and ClinVar databases is prioritized. The pathogenicity assessment of genetic variations followed the Sequence Variation Interpretation Standards and Guidelines published by the American Society of Medical Genetics and Genomics (ACMG) and used the HGVS nomenclature.

Sanger sequencing was utilized to validate the potential clinically relevant gene variations in the probands and their parents. The primer sets for PCR were designed to target gene variants using Primer Premier v5.0 software. PCR amplification was carried out using AmpliTaq Gold^*^ 360 DNA polymerase (Applied Biosystems, Shanghai, China). PCR products were then purified and sequenced on an ABI Prism 3700 automated sequencer (Applied Biosystems, CA, United States).

### Statistical analysis

Diagnostic yield was calculated based on the counts of variants classified as “pathogenic” or “likely pathogenic.” Two-sided Fisher’s test was used to test the significance of the variants. The results were considered statistically significant when the *p*-value was <.05 and the confidence interval was 95%.

## Result

### Patients’ characteristics

In total, 149 patients enrolled in the study were Han Chinese from 149 unrelated families, including 142 (95.3%) probands with urinalysis abnormalities presenting with hematuria or/and proteinuria and 7 (4.7%) patients with structural abnormalities in kidneys detected by ultrasound. They consisted of 95 (63.8%) male and 54 (36.2%) female patients with a median age of 3 years ranging from 4 days to 13 years. In total, 44 (29.5%) probands had a family history related to kidney disease (Table 1).

**TABLE 1:** Clinical details of 149 patients with suspected IKDs.

Patients	Gender	Age of onset	Protein in urine	Blood in urine	Extrarenal clinical manifestations	Family history	Clinical diagnosis
N105	F	7.6 y	3+	1+	Hypoproteinemia and hypercholesterolemia	—	NS
N184	M	4 y	3+	2+	—	Mother	AS
N191	M	6.4 y	1+	3+	Anemia	Mother	AS
N200	M	1.1 y	3+	3+	Edema, anemia, and hypercholesterolemia	—	NS
N215	M	3.7 y	2+	—	Edema, hypoproteinemia, and hypercholesterolemia	—	NS
N216	M	3.5 y	1+	—	—	—	Unknown
N221	M	7.7 y	3+	+-	Hypoproteinemia and hypercholesterolemia	—	NS
N228	F	3.5 y	2+	2+	—	—	AS
N229	F	7.6 y	—	2+	—	Father	AS
N231	M	1.4 y	3+	2+	Edema, hypoproteinemia, and hypercholesterolemia	—	NS
N232	M	11.4 y	2+	1+	Congenital cataract, short stature, and intellectual disability		Unknown
N234	F	7.5 y	—	2+	—	Mother	Unknown
N242	F	0.5 y	3+	—	Edema, hypoproteinemia, and hypercholesterolemia	—	NS
N243	F	1.2 y	3+	+-	Edema, hypoproteinemia, and hypercholesterolemia	mother	NS
N248-1	M	6.5 y	4+	—	Edema, anemia, hypoproteinemia, and hypercholesterolemia	—	NS
N259	F	—	—	1+	—	—	AS
N266	F	1.2 y	3+	—	Edema, hypoproteinemia, and hypercholesterolemia	—	Unknown
N282	M	1.8 y	3+	—	Edema, hypoproteinemia, and hypercholesterolemia	—	NS
N283	M	0.3 y	+-	—	—	—	Unknown
N284	M	1 y	4+	+-	Edema, vomiting, hypercholesterolemia, and hypoproteinemia	—	NS
N285	M	3.4 y	3+	3+	Edema, hypoproteinemia, hypercholesterolemia, and anemia	—	NS
N344	M	1 y	3+	2+	Edema, hypoproteinemia, and hypercholesterolemia	—	NS
N356	M	0.5 y	3+	3+	Diarrhea, edema, hypoproteinemia, and hypercholesterolemia, anemia	—	NS
N373	M	6 y	2+	3+	—	—	AS
N385	F	1.8 y	+-	—	Edema, hypoproteinemia, hypercholesterolemia	—	NS
N399	M	(18 days)	1+	—	Anemia and enorchia	—	NS
N419	M	8 y	+-	—	—	—	Unknown
N437	F	2 y	—	3+	—	—	Unknown
N441	M	1 y	4+	1+	Edema, cough, hypoproteinemia, and hypercholesterolemia	—	NS
N455	M	(25 days)	+	+	Cataract and jaundice	—	Unknown
N473	F	8 y	—	3+	Urinary incontinence	Father	AS
N493	F	3 y	+-	2+	Cough	Mother	Unknown
N494	M	0.7 y	2+	—	Edema, cough, anemia, hypoproteinemia, and hypercholesterolemia	—	NS
N530	M	3 y	1+	3+	Anemia	Mother	AS
N531	M	8 y	3+	3+	Psychomotor development	—	Unknown
N550	M	11 y	1+	3+	Celiodynia	Mother	AS
N587	M	3 y	2+	3+	Anemia	Mother	AS
N625	M	2.7 y	—	3+	—	Grandmother	AS
N641	M	8.8 y	3+	3+	Anemia	—	AS
N650	F	0.7 y	3+	—	Edema, hypoproteinemia, and hypercholesterolemia	—	NS
N658	F	2.3 y	2+	—	Erythema	—	NS
N668	M	9.6 y	—	2+	—	Father	Unknown
N703	M	(10 days)	4+	3+	Edema, hypoproteinemia, hypocalcemia, and poor response	—	NS
N717	F	8.4 y	4+	—	Skin rashes, edema, hypoproteinemia, and hypercholesterolemia	—	Unknown
N752	F	9.3 y	2+	3+	—	Father	Unknown
N771	M	3 y	1+	3+	Psychomotor development	Grandfather	AS
N780	M	1 y	4+	—	Edema, hypoproteinemia, and hypercholesterolemia	—	Unknown
N809	M	1 y	—	3+	—	—	AS
N810	M	1 y	3+	—	Edema, hypoproteinemia, and hypercholesterolemia	—	NS
N826	F	3.2 y	—	2+	—	—	AS
N851	M	3.9 y	—	2+	—	Father	AS
N856	F	1.8 y	—	2+	High-frequency hearing abnormalities	—	AS
N864	M	4.4 y	2+	—	edema	Brother	Unknown
N939	M	3.3 y	—	1+	—	Father	AS
N957	M	2.4 y	3+	—	Edema, hypoproteinemia, and hypercholesterolemia	—	NS
N961	M	0.7 y	2+	—	Edema	—	Unknown
N992	M	2 y	3+	—	Edema, hypoproteinemia, and hypercholesterolemia	—	NS
N1031	M	3 y	2+	2+	—	—	Unknown
N1032	M	1 y	1+	3+	—	—	AS
N1036	F	5.9 y	3+	3+	Edema, hypoproteinemia, edema, hypoproteinemia, and hypercholesterolemia	Sister	NS
N1043	M	6 y	3+	3+	Growth delay and lower level of IgM and IgG	—	Unknown
N1088	F	2.8 y	2+	3+	Skin rashes	—	Unknown
N1096	F	1.5 y	—	3+	—	Father and mother	Unknown
N1099	F	6 y	—	3+	—	Mother	AS
N1102	F	10 y	2+	2+	—	—	Unknown
N1183	M	2.8 y	2+	—	—	—	Unknown
N1218	F	5 y	3+	—	Edema, anemia, hypercholesterolemia, and hypoproteinemia	—	NS
N1238	M	2.5 y	1+	3+	—	Maternal grandmother	Unknown
N3117	M	5.7 y	3+	1+		Mother	NS
N1360	F	1 y	2+	2+	Diarrhea and convulsions	—	Unknown
N1393	M	10.6 y	2+	—		—	Unknown
N1433	M	4.5 y	1+	3+	—	Mother	Unknown
N1446	F	0.1 y	3+	1+	Convulsions, edema, convulsion, anemia, and hypoproteinemia	—	NS
N1454	M	3.9 y	+-	—	—	—	Unknown
N1477	F	1.4 y	2+	3+	—	Father	Unknown
N1492	M	5 y	2+	—	—	—	Unknown
N1497	M	(4 days)	4+	3+	Edema, hypoproteinemia, hypercholesterolemia, and hypothyroidism	—	NS
N1504	M	6.6 y	2+	1+	—	—	Unknown
N1509	M	1.9 y	4+	1+	Edema, hypercholesterolemia, and hypoproteinemia	—	NS
N1510	M	2 y	-	3+	—	—	Unknown
N1564	M	10.6 y	3+	3+	Nausea, vomiting, poor appetite, anemia and, hypoproteinemia	—	NS
N1619	M	1.9 y	3+	—	Edema, hypoproteinemia, and hypercholesterolemia	—	SRNS
N1654	M	0.9 y	2+	2+	Anemia	—	AS
N1716	F	3.7 y	-	3+	—	Father	AS
N1718	F	11.9 y	3+	3+	Anemia and nervous deafness	Mother	AS
N1735	F	1.9 y	3+	—	Edema, hypoproteinemia, and hypercholesterolemia	—	NS
N1736	M	7.8 y	3+	3+	Edema, hypoproteinemia, and hypercholesterolemia	—	NS
N1785	M	0.9 y	4+	1+	Edema, hypoproteinemia, and high-frequency hearing abnormalities	—	NS
N1786	M	6 y	2+	3+	—	—	Unknown
N1789	F	2.5 y	4+	—	Edema, hypercholesterolemia, and hypoproteinemia	—	NS
N1808	F	4.5 y	4+	—	Edema, hypoproteinemia, and hypercholesterolemia	Maternal grandmother	NS
N1823	M	7.3 y	3+	3+	Anemia	mother	AS
N1882	F	2.6 y	+-	2+	—	mother	AS
N1894	M	2.7 y	3+	3+	—	Mother	Unknown
N1927	F	5.1 y	—	1+	—	Father	AS
N1982	M	8 y	3+	—	Short stature	—	NS
N1983	F	3.5 y	1+	1+	—	Mother	Unknown
N2083	M	7.7 y	3+	1+	Edema, hypoproteinemia, and hypercholesterolemia	—	NS
N2107	F	3 y	—	3+	—	Mother	Unknown
N2135	F	7.5 y	1+		Poor eyesight	—	Unknown
N2150	F	2.9 y	2+	3+	—	Mother	Unknown
N2176	M	2.6 y	+-	3+	—	—	Unknown
N2228	M	3.3 y	—	2+	—	—	AS
N2239	F	3.9 y	—	1+	—	Mother	Unknown
N2240	M	1 y	3+	+-	Edema, hypoproteinemia, anemia, and hypercholesterolemia	—	NS
N2260	M	8 y	1+	3+	Ametropia	—	AS
N2272	M	1 y	4+	3+	Edema, hypoproteinemia, anemia, and hypercholesterolemia	—	NS
N2280	M	2 y	3+	2+	Edema, hypoproteinemia, anemia, and hypercholesterolemia	—	NS
N2290	M	4 y	3+	—	—	—	Unknown
N2320	M	1.4 y	2+	—	—	—	Unknown
N2336	M	5.5 y	2+	3+	—	—	Unknown
N2364	M	0.6 y	4+	+-	Edema and hypoproteinemia	—	NS
					Hypercholesterolemia		
N2369	M	1 y	—	1+	—	Mother	AS
N2380	M	2.3 y	4+	1+	Skin rashes, edema, hypoproteinemia, anemia, and hypercholesterolemia	—	NS
N2383	F	7 y	—	3+	Dysphagia	—	AS
N2385	M	4.7 y	3+	1+	—	—	AS
N2422	F	1.3 y	3+	2+	Edema, hypoproteinemia, hypercholesterolemia, and anemia	—	NS
N2434	M	13 y	+-	3+	Hearing abnormalities	—	AS
N2436	M	6.3 y	1+	+-	Edema, hypoproteinemia, and hypercholesterolemia	—	NS
N2489	F	4.4 y	4+	—	High-frequency hearing abnormalities, short stature, hypoproteinemia, and hypercholesterolemia	—	NS
N2562	M	2.1 y	3+	—	Language retardation, edema, hypoproteinemia, and hypercholesterolemia	—	NS
N2566	F	6 y	2+	3+	Strabismus	—	AS
N2571	M	2.4 y	2+	—	Edema, hypoproteinemia, and hypercholesterolemia	—	NS
N2628	M	11.7 y	+-	3+	—	—	AS
N2641	F	3 y	1+	3+	—	—	AS
N2718	F	6 y	2+	3+	—	Father	Unknown
N2739	F	2.1 y	2+	—	—	Maternal grandmother	AS
N2747	M	2.7 y	4+	—	Blepharoptosis in right eye	—	NS
N2769	F	1.6 y	4+	—	Edema, hypoproteinemia, and hypercholesterolemia	—	NS
N2776	F	2.9 y	+-	1+	—	—	Unknown
N2808	M	(10 days)	2+	3+	Skin rashes and aurigo	—	Unknown
N2813	F	1.6 y	3+	—	Edema, hypoproteinemia, and hypercholesterolemia	—	NS
N2849	M	1.6 y	2+	—	—	—	Unknown
N2891	M	1.8 y	—	3+	—	Mother	AS
N2899	M	1.7 y	4+	1+	Edema, hypoproteinemia, and hypercholesterolemia	—	NS
N2972	M	13 y	1+	2+	Skin rashes	Father	Unknown
N2984	M	12 y	3+	3+	Hypoproteinemia	Brother	Unknown
					Hypercholesterolemia		
N2987	F	6.7 y	3+	1+	Edema and hypoproteinemia	Mother	NS
					Hypercholesterolemia		
N2990	M	2.4 y	—	2+	—	Mother and maternal grandmother	AS
N3031	F	1.8 y	4+	1+	Edema and hypoproteinemia	—	NS
					Hypercholesterolemia		
N3253	F	5.8 y	—	3+	—	—	AS
N3295	M	3.3 y	1+	-	Edema and hypoproteinemia	—	NS
					Hypercholesterolemia and anemia		
N297	M	0.1 y			—	Mother	PKD
N482	F	6 y	1+	—	—	Mother	PKD
N855	F	0.3 y	—	2+	—	—	PKD
N2074	M	6 d			—	—	PKD
N2432	M	1 y			—	—	PKD
R96	M	0.3 y			—	—	PKD
R506	M	1.6 y			—	—	PKD

M, male; F, female; y, year(s); d, day(s); and “−,” absent.

### Genetic findings and diagnostic yield

Samples from the 149 families were tested, and 70 clinically diagnostic variants of 15 causal genes were discovered in 55 probands, encompassing 16 (29.1%) autosomal dominant IKDs, 16 (29.1%) autosomal recessive IKDs, and 23 (41.8%) X-linked IKDs. Of the 55 probands with clinically diagnostic variants, 7 (12.7%) belonged to inherited renal structural abnormality and 48 (87.3%) were inherited renal dysfunction. Diagnostic variants were found in 20 of the 32 patients (62.5%) who had a family history of kidney diseases, which was higher than those without family history: 35 diagnostic variants in the 117 patients (29.9%). The diagnostic yield was 36.9% (Table 2; Table 3).

**TABLE 2:** Variants analyzed by exome sequencing in the patients from 55 Chinese families.

Patient	Sex	Age of onset	Diagnosis	Inheritance	*gene*	Exon	DNA change	Effect	Mutation type	Status	Variation origin	Pathogenic evaluation according to ACMG
N184	M	4 y	XLAS	XD	*COL4A5*	32	c.2731G>T	p.G911X	Non-sense	Novel	Maternal	LP
N191	M	6.4 y	XLAS	XD	*COL4A5*	34	c.2990_2991insGCAACC	p.G997delinsGQPGLSV	indel	Novel	Maternal	LP
							TGGACTGAGTGT					
N229	F	7.6 y	XLAS	XD	*COL4A5*	47	c.4352G>A	p.G1451D	Missense	Reported	Paternal	P
N259	F	13 y	XLAS	XD	*COL4A5*	47	c.4515 + 1G>A	Splicing	Splicing	Reported	NA	LP
N473	F	8 y	XLAS	XD	*COL4A5*	9	c.466G>C	p.G156R	Missense	Reported	Paternal	LP
N530	M	3 y	XLAS	XD	*COL4A5*	13	c.689G>T	p.G230V	Missense	Reported	Maternal	P
N550	M	11 y	XLAS	XD	*COL4A5*	20	c.1226G>C	p.G409A	Missense	Reported	Maternal	LP
N587	M	3 y	XLAS	XD	*COL4A5*	29	c.2314G>C	p.G772R	Missense	Novel	Maternal	LP
N752	F	9.3 y	XLAS	XD	*COL4A5*	47	c.4324dupG	p.P1443Sfs*43	Frameshift	Novel	Paternal	P
N1716	F	3.7 y	XLAS	XD	*COL4A5*	3	c.231 + 1G>T		Splicing	Reported	Paternal	LP
N1823	M	7.3 y	XLAS	XD	*COL4A5*	31	c.2605G>A	p.G869R	Missense	Reported	Maternal	P
N1894	M	2.7 y	XLAS	XD	*COL4A5*	51	c.5029C>T	p.R1677X	Non-sense	Reported	Maternal	LP
N1927	F	5.1 y	XLAS	XD	*COL4A5*	19	c.1033–6A>G		Splicing	Reported	Paternal	LP
N1983	F	3.5 y	XLAS	XD	*COL4A5*	19	c.1114G>T	p.E372X	Non-sense	Novel	Maternal	P
N2150	F	2.9 y	XLAS	XD	*COL4A5*	1–26	exon1-26 DEL		exon del	Reported	NA	P
N2566	F	6 y	XLAS	XD	*COL4A5*	25	c.1913G>T	p.G638V	Missense	Reported	*De novo*	P
N2718	F	6 y	XLAS	XD	*COL4A5*	23	c.1562G>A	p.G521D	Missense	Reported	Paternal	P
N2984	M	12 y	XLAS	XD	*COL4A5*	35	c.3106 + 1G>A		Splicing	Reported	*De novo*	P
N3253	F	5.8 y	XLAS	XD	*COL4A5*	19	c.1117C>T	p.R373X	Non-sense	Reported	*De novo*	P
N668	M	9.6 y	ADAS	AD	*COL4A3*	11	c.620G>A	p.G207E	Missense	Novel	Paternal	LP
N851	M	3.9 y	ADAS	AD	*COL4A3*	40	c.3499G>C	p.G1167R	Missense	Novel	Paternal	LP
N1433	M	4.5 y	ADAS	AD	*COL4A3*	25	c.1622G>A	p.G541D	Missense	Novel	Maternal	LP
N1882	F	2.6 y	ADAS	AD	*COL4A3*	47	c.4216G>A	p.G1406R	Missense	Reported	Maternal	LP
N2628	M	11.7 y	ADAS	AD	*COL4A3*	37	c.3098G>T	p.G1033V	Missense	Reported	*De novo*	LP
N1477	F	1.4 y	ADAS	AD	*COL4A3*	34	c.2806C>T	p.Q936X	Non-sense	Reported	Paternal	P
N1099	F	6 y	ADAS	AD	*COL4A3*	46	c.4136G>A	p.G1379E	Missense	Novel	Maternal	LP
N2990	M	2.4 y	ADAS	AD	*COL4A4*	35	c.1951G>C	p.G651R	Missense	Novel	Maternal	LP
N234	F	7.5 y	ADAS	AD	*COL4A4*	48	c.5049C>A	p.C1683X	Non-sense	Novel	Maternal	LP
N493	F	3 y	ADAS	AD	*COL4A4*	47	c.4788G>A	p.W1596X	Non-sense	Reported	Maternal	LP
N1096	F	1.5 y	ARAS	AR	*COL4A4*	17	c.1022G>A	p.G341D	Missense	Novel	Paternal	LP
						14	c.870G>A	Splicing	Splicing	Reported	Maternal	LP
N1718	F	11.9 y	ARAS	AR	*COL4A4*		2q36.3 (chr2:227729409–227959054)*1		Deletion		NA	LP
						38	c.3506-13_3528del	p.G1169Efs*13	Frameshift	Reported	Maternal	LP
N1036	F	5.9 y	ARAS	AR	*COL4A3*	51	c.4793T>G	p.L1598R	Missense	Reported	Maternal	LP
						25	c.1604G>A	p.G535E	Missense	Novel	Paternal	LP
N703	M	(10 days)	NPHS1	AR	*NPHS1*	28	c.3555delG	p.V1186Sfs*52	Frameshift	Novel	Maternal	P
						27	c.3478C>T	p.R1160X	Non-sense	Reported	Paternal	LP
N1446	F	0.1 y	NPHS1	AR	*NPHS1*	16	c.2207T>C	p.V736A	Missense	Reported	Maternal	LP
						16	c.2210A>C	p.H737P	Missense	Novel	Paternal	LP
N1497	M	(4 days)	NPHS1	AR	*NPHS1*	11	c.1338delT	p.I446fs*16	Frameshift	Reported	Maternal	LP
						9	c.1103C>T	p.P368L	Missense	Reported	Paternal	P
N3031	F	1.8 y	NPHS1	AR	*NPHS1*	2	c.223C>T	p.P75S	Missense	Novel	Paternal	LP
						1	c.14C>T	p.T5M	Missense	Reported	Maternal	LP
N1982	M	8 y	SIOD	AR	*SMARCAL1*	16	c.2459G>A (hom)	p.R820H	Missense	Reported	Maternal, paternal	P
N2489	F	4.4 y	SIOD	AR	*SMARCAL1*	7	c.1334 + 1G>A		Splicing	Reported	Maternal	P
						5	c.971dupC	p.A325Sfs*34	Frameshift	Novel	Paternal	P
N216	M	3.5 y	DENT1	XR	*CLCN5*	11	c.1444delG	p.G483Vfs*21	Frameshift	Novel	Maternal	LP
N1504	M	6.6 y	DENT1	XR	*CLCN5*	7	c.773C>G	p.P258R	Missense	Novel	Maternal	LP
N399	M	(18 days)	DENT2	XR	*OCRL*	12	c.1178delT	p.I393fs	Frameshift	Novel	Maternal	LP
N232	M	11.4 y	OCRL	XR	*OCRL*	18	c.1978C>T	p.H660Y	Missense	Novel	Maternal	LP
N3117	M	5.7 y	FSGS2	AD	*TRPC6*	8	c.2170delA	p.I724Lfs*3	Frameshift	Novel	Maternal	LP
N243	F	1.2 y	FSGS5	AD	*INF2*	8	c.1581dupC	p.V530Rfs*50	Frameshift	Reported	Maternal	LP
N1564	M	10.6 y	FSGS7	AD	*PAX2*	3	c.226G>A	p.G76S	Missense	Reported	*De novo*	P
N105	F	7.6 y	FSGS9	AR	*CRB2*	8	c.2277G>A	p.W759X	Non-sense	Reported	Maternal	P
						10	c.3190C>T	p.P1064S	Missense	Reported	Paternal	LP
N1043	M	6 y	ILFS2	AR	*NBAS*	25	c.2882T>A	p.L961X	Non-sense	Novel	Maternal	LP
						52	c.7041_7043delTCT	p.2347_2348delLLinsL	Indel	Reported	Paternal	LP
N1785	M	0.9 y	NPHS12	AR	*NUP93*	6–7	exon6-7 Del		Exon del	Reported	NA	LP
						16	c.1772G>T	p.G591V	Missense	Reported	Maternal	LP
N855	F	0.3 y	ARPKD	AR	*PKHD1*	21	c.1981A>C	p.T661P	Missense	Reported	Paternal	LP
						3	c.55C>T	p.R19C	Missense	Reported	Maternal	LP
N2074	M	6 d	ARPKD	AR	*PKHD1*	13	c.920T>C	p.I307T	Missense	Reported	Maternal	LP
						50	c.7994T>C	p.L2665P	Missense	Reported	Paternal	LP
R96	M	0.3 y	ARPKD	AR	*PKHD1*		c.3500T>C	p.L1167P	Missense	Novel	Maternal	LP
							c.9235_9236delGCinsAA	p.A3079K	Missense	Novel	Paternal	LP
R506	M	1.7 y	ARPKD	AR	*PKHD1*	60	c.10756_10759delAACT	p.N3586Sfs*22	Frameshift	Reported	Paternal	LP
						26	c.2876C>T	p.S959F	Missense	Reported	Maternal	LP
N297	M	0.1 y	ADPKD	AD	*PKD1*	15	c.6070C>T	p.R2024C	Missense	Reported	Maternal	LP
N482	F	6 y	ADPKD	AD	*PKD1*	15	c.4759C>T	p.R1587C	Missense	Reported	Maternal	LP
N2432	M	1 y	ADPKD	AD	*PKD1*	15	c.5218_5220delCACCCTCAGTGCCinsTT	p.T1737fs*	Frameshift	Reported	*De novo*	P

M, male; F, female; y, year(s); d, day(s); XLAS, X-linked Alport syndrome; ADAS, autosomal dominant Alport syndrome; ARAS, autosomal recessive Alport syndrome; NPHS1, nephrotic syndrome type 1; SIOD, Schimke immunoosseous dysplasia; DENT1, Dent disease 1; DENT2, Dent disease 2; OCRL, Lowe oculocerebrorenal syndrome; FSGS2, glomerulosclerosis focal segmental 2; FSGS5, glomerulosclerosis focal segmental 5; FSGS7, glomerulosclerosis focal segmental 7; FSGS9, glomerulosclerosis focal segmental 9; ILFS2, infantile liver failure syndrome-2; NPHS12, nephrotic syndrome type 12; XD, X-linked dominant; XR, X-linked recessive; AD, autosomal dominant; AR, autosomal recessive.

**TABLE 3:** Evaluation of novel variants according to the ACMG guidelines.

Patient	Gene	DNA change	Effect	ACMG	
N184	*COL4A5*	c.2731G>T	p.G911X	PVS1, PM2, PP3, PP4	LP
N191	*COL4A5*	c.2990_2991insGCAACCTGGACTGAGTGT	p.G997delinsGQPGLSV	PM1, PM2, PP1, PP3, PP4	LP
N216	*CLCN5*	c.1444delG	p.G483Vfs*21	PVS1, PM2	LP
N232	*OCRL*	c.1978C>T	p.H660Y	PM2, PM5, PP1, PP3, PP4	LP
N3117	*TRPC6*	c.2170delA	p.I724Lfs*3	PVS1, PM5	LP
N234	*COL4A4*	c.5049C>A	p.C1683X	PVS1, PM2, PP4	LP
N399	*OCRL*	c.1178delT	p.I393fs	PVS1, PM2, PP4	LP
N587	*COL4A5*	c.2314G>C	p.G772R	PM2, PM5, PP1, PP3, PP4	LP
N668	*COL4A3*	c.620G>A	p.G207E	PM2, PP1, PP2, PP3, PP4	LP
N703	*NPHS1*	c.3555delG	p.V1186Sfs*52	PVS1, PM2, PM3	P
N752	*COL4A5*	c.4324dupG	p.P1443Sfs*43	PVS1, PM2	P
N851	*COL4A3*	c.3499G>C	p.G1167R	PS1, PP2, PP1, PP3	LP
N1036	*COL4A3*	c.1604G>A	p.G535E	PM2, PM3, PP3, PP4	LP
N1043	*NBAS*	c.2882T>A	p.L961X	PVS1, PM2	LP
N1096	*COL4A4*	c.1022G>A	p.G341D	PM1, PM2, PP3, PP4	LP
N1433	*COL4A3*	c.1622G>A	p.G541D	PM2, PP1, PP2, PP3, PP4	LP
N1446	*NPHS1*	c.2210A>C	p.H737P	PM1, PM2, PM3	LP
N1504	*CLCN5*	c.773C>G	p.P258R	PM1, PM2, PP3, PP4	LP
N1983	*COL4A5*	c.1114G>T	p.E372X	PVS1, PM2, PP1	P
N2489	*SMARCAL1*	c.971dupC	p.A325Sfs*34	PSV1, PM2, PM3	P
N2990	*COL4A4*	c.1951G>C	p.G651R	PM2, PP1, PP2, PP3, PP4	LP
N1099	*COL4A3*	c.4136G>A	p.G1379E	PM2, PP1, PP2, PP3, PP4	LP
N3031	*NPHS1*	c.223C>T	p.P75S	PM1, PM2, PM3, PP4	LP
R96	*PKHD1*	c.3500T>C	p.L1167P	PM2, PM3, PP4	VUS
R96	*PKHD1*	c.9235_9236delGCinsAA	p.A3079K	PM1, PM2, PP3, PP4	LP

P, pathogenic; LP, likely pathogenic; VUS, variant of unknown significance.

The seven probands with inherited renal structural abnormality included four autosomal recessive polycystic kidney disease (ARPKD) caused by the compound heterozygous variants in *PKHD1* and three autosomal dominant polycystic kidney disease (ADPKD) caused by the heterozygous variants in *PKD1*.

Of the 48 probands with inherited renal dysfunction, 32 were Alport syndrome (AS) caused by the variants in the genes of *COL4A5*, *COL4A3*, or *COL4A4* with different inheritance patterns; four inherited nephrotic syndrome type 1 (NPHS1) caused by the compound heterozygous variants in the gene of *NPHS1*, two Schimke immunoosseous dysplasia (SIOD) caused by the homozygous or compound heterozygous variants in the *SMARCAL1* gene, two Dent disease type 1 (DENT1) caused by the heterozygous variants in the *CLCN5* gene, one Dent disease type 2 (DENT2) caused by the heterozygous variant in the gene of *OCRL*, one Lowe oculocerebrorenal syndrome (OCRL) caused by the heterozygous variant in the *OCRL* gene, one glomerulosclerosis focal segmental 2 (FSGS2) caused by the heterozygous variant in the gene of *TRPC6*, one glomerulosclerosis focal segmental 5 (FSGS5) caused by the heterozygous variant in the gene of *INF2*, one glomerulosclerosis focal segmental 7 (FSGS7) caused by the heterozygous variant in the gene of *PAX2*, one focal segmental glomerulosclerosis 9 (FSGS9) caused by the compound heterozygous variant in the gene of *CRB2*, one infantile liver failure syndrome-2 (ILFS2) caused by the compound heterozygous variant in the gene of *NBAS*, and one nephrotic syndrome type 12 (NPSH12) caused by the compound heterozygous variant in the gene of *NUP93*.

**FIGURE 1:**
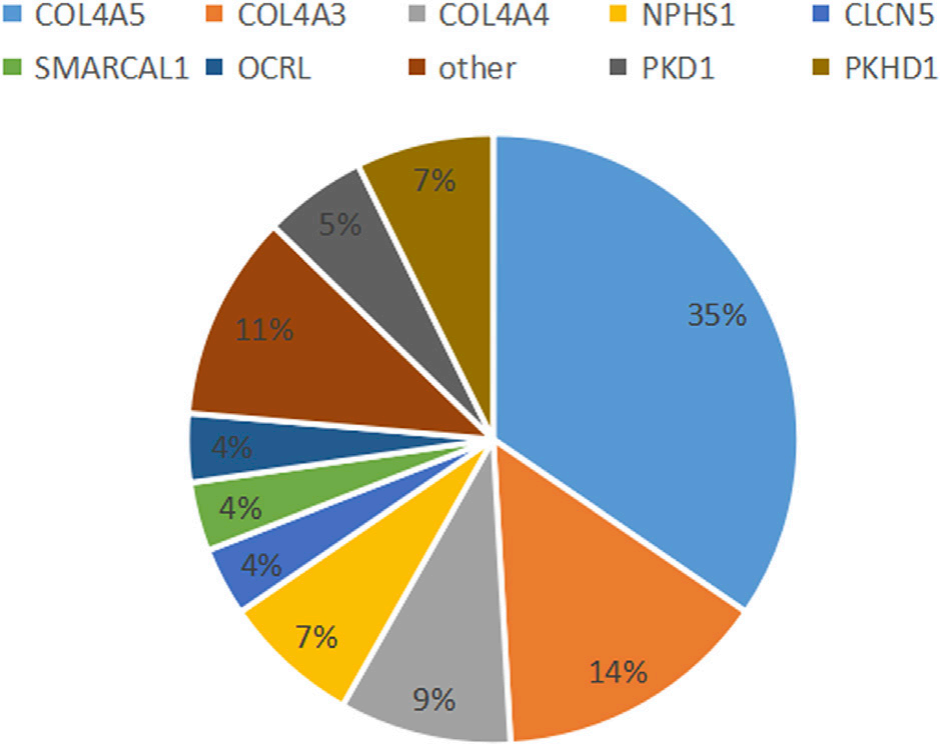
Most common diagnostic genetic findings. In total, 15 causative genes, representing 16 distinct monogenetic disorders, were detected in 55 patients.

The 70 clinically diagnostic variants of 15 causal genes comprised 11 variants in two genes leading to inherited renal structural abnormality and 59 resulting in inherited renal dysfunction in 13 genes with 25 unreported and 45 reported previously. Of the 70 variants, one was homozygous from both parents and six *de novo*, 22 parental, 37 maternal, and four unknown origin variants due to unavailable parents. The 11 variants causing seven inherited renal structural abnormalities included nine missense variants and two frameshift variants. The 59 variants resulting in inherited renal dysfunction consisted of 29 (49.2%) missense, 10 (16.9%) nonsense, 9 (15.2%) frameshift, 6 (10.2%) splicing, 2 (3.4%) indel, and 3 (5.1%) gross deletion variants (Table 2; Figure 1).

### Novel variants

Among the 55 patients with confirmed disease according to molecular diagnosis, 25 novel variants in 24 patients absent from population databases and our in-house database were identified (Table 3).

Of the 25 novel variants, 13 were found in the genes of *COL4A5*, *COL4A4*, and *COL4A3* in 16 probands with Alport syndrome (AS). There were five novel variants in the *COL4A5* gene in five probands, i.e., three male and two female patients, including one missense (p.G772R), two nonsense (p.G911X and p.E372X), one frameshift (p.P1443Sfs*43), and one indel (p.G997delinsGQPGLSV). Among the five probands, two female probands showed mild urinalysis abnormalities with no or little protein in urine, while three male probands indicated significant hematuria and proteinuria. They were genetically diagnosed with XLAS after exome sequencing. Five novel variants in the *COL4A3* gene identified in five patients were all missense variants of p.G207E, p.G1167R, p.G541D, p.G535E, and p.G1379E. Furthermore, the variant of p.G535E combined with a reported one of p.L1598R, was detected in a 5.9-year-old girl (N1036) who presented with distinct hematuria and proteinuria with level 3 originally clinically diagnosed as nephrotic syndrome (NS). Both the variants of p.G535E and p.L1598R were obtained from her father and mother, separately, which constituted a compound heterozygous mutation effect; thus, the patient was finally diagnosed with ARAS. The remaining four variants were in the *COL4A3* gene with two paternal and two maternal variation origins in other four patients who mainly presented with hematuria with strong family history, and they were diagnosed with ADAS. There were three novel variants in *COL4A4* in three probands who manifested remarkable hematuria and were genetically diagnosed with ADAS.

Three novel variants in the *NPHS1* gene were identified as one frameshift (p.V1186Sfs*52) in Patient N703 and two missense (p.H737P and p.P75S) in patients N1446 and N3031. Three patients were very young, presenting with severe proteinuria with primary diagnosis of NS, and were finally confirmed as having nephrotic syndrome type 1 (NPHS1).

We detected two novel variants in the *OCRL* gene in two probands. Patient N399, an 18-day-old boy, showed recurrent proteinuria, and his genetic investigation revealed a maternally inherited variant (p.I393fs) in the *OCRL* gene. He was diagnosed with DENT2 after genetic testing, whereas the other was a maternally inherited missense variant (p.H660Y) in patient N232, who presented with hematuria, proteinuria, and extrarenal symptoms of congenital cataract, short stature, and intellectual disability that were consistent with the Lowe oculocerebrorenal syndrome (OCRL).

Other seven novel variants were identified in five genes, namely, *SMARCAL1*, *CLCN5*, *NBAS*, *TRPC6*, and *PKHD1* in six probands, who were diagnosed as SIOD, DENT1, ILFS2, FSGS2, and ARPKD (Table 3).

### Clinical implications of genetic diagnosis

**FIGURE 2:**
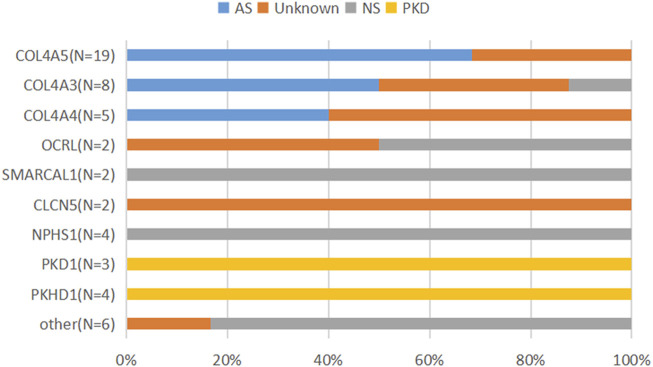
Clinical diagnostic spectrum of patients with genetic diagnosis of IKDs. The patients who were clinically diagnosed with NS were associated with *CLO4A3*, *OCRL*, *SMARCAL1*, and *NPHS1* or other genes; the patients who were clinically diagnosed with AS were associated with *COL4A3*, *COL4A4*, and *COL4A5*; the patients clinically undiagnosed (unknown) were associated with the genes of *COL4A5*, *COL4A3*, *COL4A4*, *OCRL*, and *CLCN5* and others.

**FIGURE 3:**
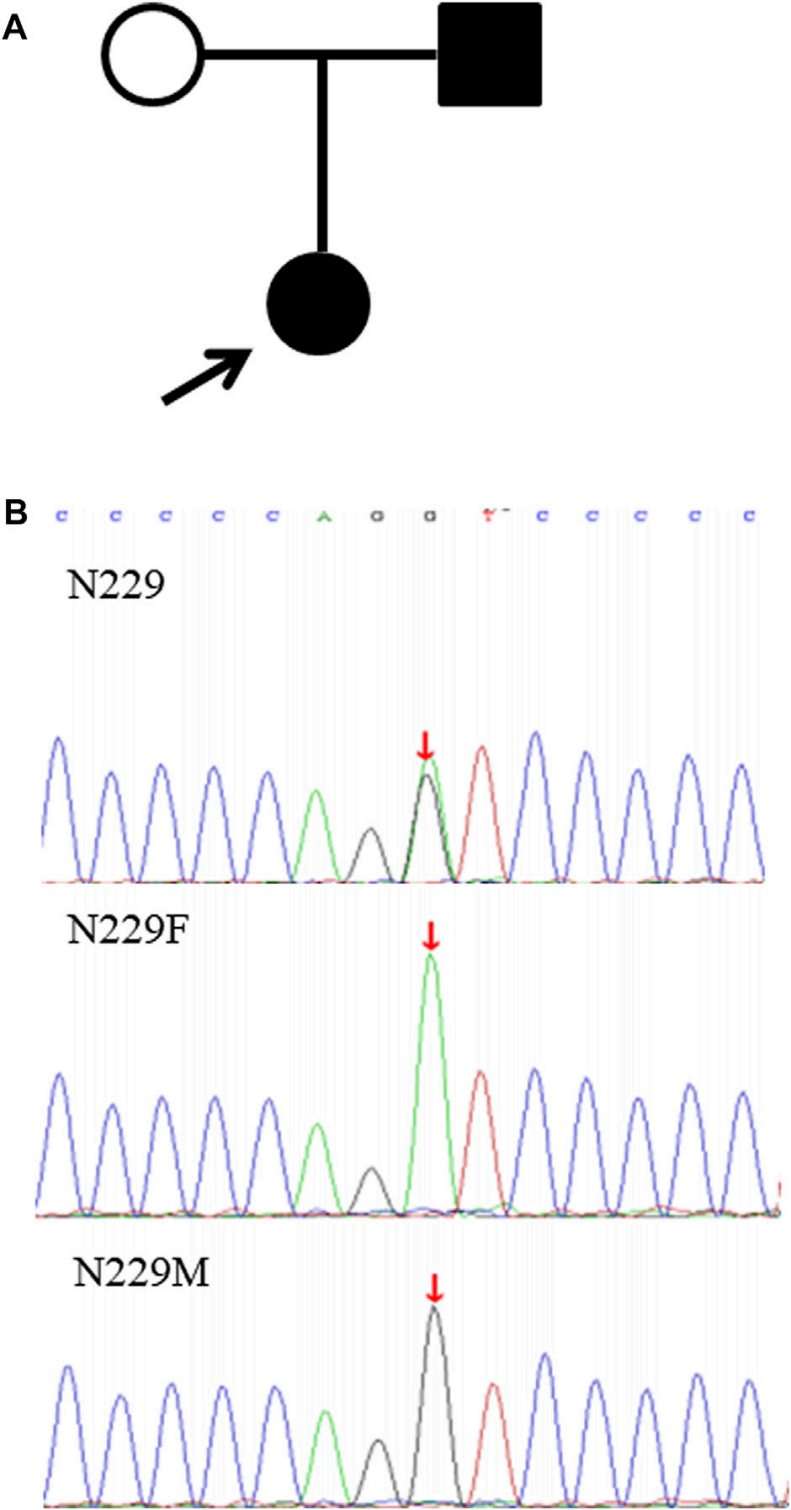
Pedigree chart and verification of the variant in *COL4A5* in the N229 family. **(A)** Pedigree chart of the N229 family; **(B)** the variant c.4352G>A in *COL4A5* validated by Sanger sequencing in the proband (N229), father (N229F), and mother (N229M).

In this study, the 55 probands detected with causative variants by NGS were genetically diagnosed, and the clinical diagnostic utility of the exome sequencing was assessed after more detailed clinical data were analyzed. Among them, 12 patients (21.8%) were confirmed suspected IKD cases**,** 26 patients (47.3%) discerned the specific subtypes of clinical category, and 17 patients (30.9%) with unknown etiology or lack of typical manifestations were reclassified so that the specific intervention was managed in a timely manner (Table 4; Figure 2). Taking the N229 family, for instance, the proband, a 7.6-year-old girl and the only child of her parents, visited our hospital for the first time in December 2019 due to her positive urine occult blood test (level 2) and strong family history, while there were no obvious symptoms. Her 25-year-old father had suffered from uremia and was on kidney dialysis regularly. Both the patients and her father were detected the variant of c.4352G>A (p.G1451D) in the *COL4A5* gene and finally diagnosed with XLAS (Figure 3). From then on, the patient has received enalapril maleate tablet (angiotensin-converting enzyme inhibitor) therapy, plus regular urine test for three years, demonstrating a good condition without proteinuria since then.

**TABLE 4:** Diagnostic utility in 55 patients with genetic diagnosis of IKDs.

	Confirmed suspected disease	Discerned specific subtype	Reclassified disease
Patients, n (%)	12 (21.8%)	26 (47.3%)	17 (30.9%)
Distinct genetic disorder^a^	7	5	7
Singleton case^b^	6	0	2

^a^ Encompassing 17 distinct monogenic disorders.

^b^ A total of eight genetic disorders were unique to a single patient.

## Discussion

Several studies indicated that NGS has particular benefits for children with IKDs by Providing early diagnosis, which would affect follow-up treatment and improve outcomes in clinical practice (Groopman et al., 2019; Zamani et al., 2021; Knoers et al., 2022; Kashtan, 2021). In the present study, we investigated the genetic cause of 149 child probands with clinically suspected IKDs or unexplained proteinuria or/and hematuria using exome sequencing. In total, 55 probands were found with clinically significant variants involving 15 causative genes and were then diagnosed with 16 monogenic disorders. Of them, 14 patients (25.5%) presenting with mild urinalysis abnormalities of trace blood or protein in urine were precisely identified so that the intervention was performed and potential extrarenal symptoms or complications could be monitored. The diagnostic yield was 36.9% (55/149), which is higher than that in Groopman’s report (Groopman et al., 2019) while is similar as two previous studies in children in two Asian populations of Seoul, South Korea, and Shanghai, South China, with the diagnostic yields of 39.2% and 39.9%, respectively (Chen et al., 2021; Oh et al., 2021), further demonstrating that exome sequencing is a powerful tool for the early genetic profiling of IKDs and genetic variability among ethnic and geographical regions.

It is reported that genetic diagnosis contributed to the reclassification of original diagnosis in 10%–22% of cases (Groopman et al., 2019; Knoers et al., 2022). In this study, a higher number of 32.7% (18/55) patients with unknown etiology or lack of typical manifestations were reclassified for which we considered two reasons: first, the patients with IKDs have heterogeneity of genotype and phenotype in different populations, particularly in children at early stages of IKDs; second, most IKDs are rare diseases, some of which are unknown even to pediatric nephrologists (Groopman et al., 2019; Murray et al., 2020; Torra et al., 2021). For example, patient N216 was a 3.5-year-old boy manifesting only little protein in urine without other specific symptoms, who presented with a potential kidney disease of an unknown cause until the genetic result was received.

Genetic testing plays a crucial role in precise genetic counseling by providing information about hereditary modes, accessing recurrence risks, and avoiding giving birth to an affected child with the same disease (de Haan et al., 2019; Wang and Weng., 2017). For instance, Alport syndrome (AS) is a progressive hereditary renal disease accompanied by sensorineural hearing loss and ocular abnormalities, caused by the pathogenic variants in the genes of *COL4A3*, *COL4A4*, and *COL4A5* encoding type IV collagen α3, α4, and α5 chains, respectively (Nozu et al., 2020; Kashtan, 2021). In this study, 32 probands with AS were reclassified into specific subtypes of 19 X-linked Alport syndrome (XLAS), 10 autosomal dominant Alport syndrome (ADAS), and three autosomal recessive Alport syndrome (ARAS) based on the exome sequencing. In total, of 35 variants found in 32 probands, only four variants were *de novo*. Of the 32 probands, 22 had a family history, with four parents presenting with uremia or renal failure. Hence, genetic counseling is particularly essential for families to perform prenatal diagnosis in subsequent pregnancies.

Alport syndrome (AS) has been reported as the second most common IKD following ADPKD in Europe and the US during adulthood (Groopman et al., 2019; Oberdhan et al., 2022), with most of them (about 80%) being XLAS caused by the pathogenic variants in the *COL4A5* gene (Nozu et al., 2020), which leads to heterogeneous renal manifestations from hematuria alone to end-stage kidney disease. In male patients with XLAS, there is an evident genotype–phenotype correlation: patients with nonsense variants having the most severe phenotypes, patients with splicing variants having moderate phenotypes, and patients with missense variants presenting with mild phenotypes (Aoto et al., 2022). Our study showed that AS was the most common IKD in children in Shandong, China, and the percentage of patients with XLAS was 59.4% (19/32), which was lower than that found in the previous report. As the most common variants were missense, the patients with AS often presented with mild manifestations without extrarenal phenotypes, which could lead to the parents or even the clinicians failing to recognize it in a timely manner. This might be corresponding to the lower percentage of AS patients with the *COL4A5* variant.

Exome sequencing is demonstrated to be a powerful genetic tool in clinical practice settings, but there are still some limitations. The introns and promoter regions of genes were not included in the exome sequencing carried out in our study, and the variants in low coverage areas of exons might be filtered out. In addition, new genes of unknown function might be found in these patients. Finally, HLA-DR/DQ alleles and haplotypes might be associated with some patients. All of the aforementioned factors need to be explored in the future.

In conclusion, nearly 36.9% (55/149) of children with abnormal kidney structure or function received a precise diagnosis by genetic testing. AS is the most common disease in Chinese children, mostly presenting with mild symptoms, and 25 novel variants in 24 patients were identified, which expands the variant spectrum of causative genes in IKDs. For clinical diagnostic applications in children with suspected IKDs, genetic testing could be performed to provide an accurate molecular diagnosis and more guidance in follow-up treatment and prognostic assessment.

## Data Availability

The datasets presented in this study can be found in online repositories. The names of the repository/repositories and accession number(s) can be found at: https://www.ncbi.nlm.nih.gov/genbank/,ON381715-ON381719.
